# Detection of H3F3A K27M or BRAF V600E in liquid biopsies of brain tumor patients as diagnostic and monitoring biomarker: impact of tumor localization and sampling method

**DOI:** 10.1007/s00401-024-02842-7

**Published:** 2025-01-03

**Authors:** Sibylle Madlener, Natalia Stepien, Daniel Senfter, Lisa Mayr, Anna Laemmerer, Cora Hedrich, Alicia Baumgartner, Daniela Lötsch-Gojo, Jaroslav Sterba, Petra Pokorna, Barbara Kiesel, Georg Widhalm, Franziska Eckert, Matthias Preusser, Karl Rössler, Amedeo Azizi, Andreas Peyrl, Thomas Czech, Christine Haberler, Irene Slavc, Gregor Kasprian, Christian Dorfer, Julia Furtner, Johannes Gojo

**Affiliations:** 1 Department of Pediatrics and Adolescent Medicine, Comprehensive Center for Pediatrics and Comprehensive Cancer Center, Medical University of Vienna, Vienna, Austria; 2 Center for Cancer Research, Medical University of Vienna, Vienna, Austria; 3 Department of Neurosurgery, Medical University of Vienna, Vienna, Austria; 4 Department of Pediatric Oncology, University Hospital Brno and Faculty of Medicine, Masaryk University, Brno, Czech Republic; 5 Department of Biology, Faculty of Medicine and Central, European Institute of Technology, Masaryk University, Brno, Czech Republic; 6 Center for Precision Medicine, University Hospital Brno, Brno, Czech Republic; 7 Department of Radiation Oncology, Medical University of Vienna, Vienna, Austria; 8 Department of Internal Medicine I, Medical University of Vienna, Vienna, Austria; 9 Division of Neuropathology and Neurochemistry, Department of Neurology, Medical University of Vienna, Vienna, Austria; 10 Department of Biomedical Imaging and Image-Guided Therapy, Medical University of Vienna, Vienna, Austria; 11 Research Center of Image Analysis and Artificial Intelligence (MIAAI), Faculty of Medicine and Dentistry, Danube Private University, Krems-Stein, Austria

**Keywords:** Liquid biopsy, Glioma, Longitudinal monitoring, Targeted therapy, Droplet digital PCR, CSF sampling site

## Abstract

Gliomas are the most common brain tumor type in children and adolescents. To date, diagnosis and therapy monitoring for these tumors rely on magnetic resonance imaging (MRI) and histopathological as well as molecular analyses of tumor tissue. Recently, liquid biopsies (LB) have emerged as promising tool for diagnosis and longitudinal tumor assessment potentially allowing for a more precise therapeutic management. However, the optimal strategy for monitoring gliomas by LB remains to be determined. In this study, we analyzed circulating tumor DNA (ctDNA) from 78 liquid biopsies (plasma *n* = 44, cerebrospinal fluid *n* = 34 (CSF)) of 35 glioma patients, determining H3F3A K28M (K27M) and BRAF V600E mutation allele frequency using droplet digital PCR (ddPCR). All results were correlated to clinically relevant parameters including diagnostic imaging and CSF aspiration site (ventricular vs lumbar) with respect to tumor localization. Regarding diagnostic accuracy, the calculated sensitivity score in the H3F3A K27M cohort was 84.61% for CSF and 73.68% for plasma. In the BRAF V600E cohort, we determined a sensitivity of 83.3% in plasma and 80% in CSF. The overall specificity was 100%. With respect to the CSF aspiration, the intra-operatively obtained CSF demonstrated 100% detection rate, followed by ventricular CSF obtained via Ommaya Reservoir/shunt puncture (93%) and CSF obtained via lumbar puncture (66%). Notably, this further correlated with the proximity of the CSF site to tumor localization. Longitudinal CSF monitoring demonstrated a good correlation to clinical and radiological disease evolution. Importantly, we show for the first time that monitoring BRAF V600E by ddPCR could serve as treatment response assessment in gliomas. In summary, our observation may inform recommendations with regard to location of CSF aspiration when incorporating LB into future treatment protocols.

**Supplementary Information:**

The online version contains supplementary material available at 10.1007/s00401-024-02842-7.

## Introduction

Tumors of the central nervous system (CNS) are the most frequent solid tumor type in children and adolescents [26], and molecular profiling efforts have revealed multiple diagnostic and prognostic biomarkers, already used in clinical routine [23]. Among glioma, the most frequent CNS tumor entity, mutations within canonical histones H3.1 (*HIST1H3B/C*), H3.2 (*HIST2H3C*) and non-canonical histone H3.3 (*H3F3A*) [7, 31] as well as *BRAF* are well-established markers routinely analyzed in tumor tissue. While the detection of *H3K27M* histone mutations is used to classify these tumors as diffuse midline glioma, DMG H3K27-altered and defines these tumors as CNS WHO grade 4 tumors according to the WHO 2021 brain tumor classification [23], *BRAF* alterations (gene fusions or point mutations) arise in both low-grade gliomas (LGG) and a subset of high-grade gliomas (HGG) [6, 24]. DMGs with H3K27 alteration have a dismal prognosis with a mean overall survival of 9–12 months [14, 15, 19, 29]. They arise in the midline of brain structures (thalamus, pons, spinal cord) and a gross total resection is not feasible in most cases. So far, in most cases, tumor biopsies or partial resection depending on tumor location are the only possibilities to identify the molecular biology and potential treatment strategies for DMG. Due to the delicate location, biopsies are not routinely repeated as the disease progresses, making it impossible to study the molecular tumor development and molecular treatment response. *BRAF* alterations are currently detected through analysis of tumor tissue obtained during tumor resection [36]. *BRAF* alterations occur in both LGG and HGG, the latter mostly located in either the cerebellum or supratentorial hemispheres with excellent or fatal prognosis, respectively [17]. Importantly, targeted therapy against BRAF V600E, the most common point mutation, is widely used in these patients and combination therapy with trametinib and dabrafenib has been approved in December 2023 by the European Medicines Agency for use in pediatric LGG and in relapsed BRAF V600E mutant pediatric HGG [3, 13]. However, tumors may also develop resistance to BRAF/MEK-inhibitors. Considering the sensitive tumor locations within the CNS and the molecular dynamics in the respective tumors in the course of disease, there is a compelling need to identify more specific and accessible markers for clinical use. Analysis of circulating tumor DNA (ctDNA) has emerged as a promising tool for disease monitoring in various cancer types [8, 9, 34, 35]. In the case of pediatric CNS tumors, analysis of cerebrospinal fluid (CSF) has been shown to provide a feasible source for detection of molecular alterations and some studies suggested superiority when compared to blood [18, 22, 25, 33, 39]. However, retrieval of CSF also usually requires an invasive procedure by implanting a reservoir in situ or lumbar puncture [2, 29]. Hence, more easily accessible body fluids for performing liquid biopsies (LBs) would be desirable. Moreover, highly sensitive methods have already shown that blood may also provide a feasibly source for the detection of tumor specific alterations. In our previous study, we demonstrated the potential for longitudinal molecular monitoring in a single H3F3A K27M glioma patient [12]. In this study, we present the utility of LB for diagnosis and disease monitoring of H3F3A K27M and BRAF V600E altered tumors with a follow-up up to 24 months. Supplementary Fig. 1 illustrates the study design. Importantly, our study explored for the first time whether CSF obtained via ventricular access devices (Ommaya Reservoir, ventriculo-peritoneal (VP) shunt) represents a feasible way to allow for less invasive LB analysis in pediatric glioma.

## Materials and methods

### Patient samples

**Fig. 1:**
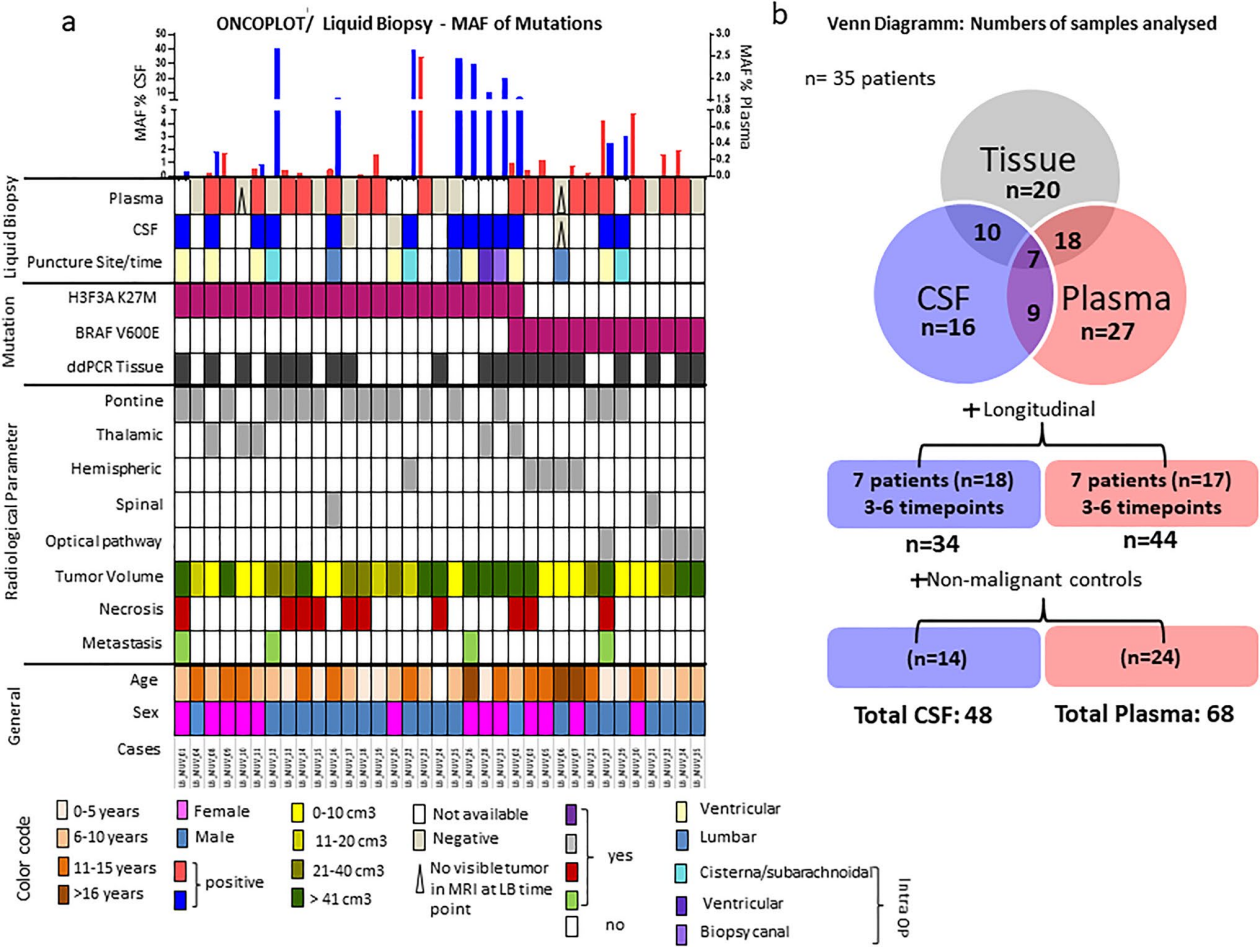
Summary of patient characteristics and overview of analyzed samples: **a** Oncoplot, **b** Venn diagram showing the number of initial samples analyzed and matching cases between the samples. In addition, the number of longitudinal cases and controls of the individual liquids are also shown

35 patients were included, 34 glioma patients across all pediatric age groups and 1 adult glioma patient with a gender ratio of 13/22 (female/male). 22 patients harbored a H3F3A K27M mutation, 12 patients a BRAF V600E mutation and 1 patient both mutations in their tumor tissue. Mutations were confirmed either with panel sequencing (TruSight500, Oncomine Childhood Cancer Research Assay or Oncomine Comprehensive Assay Version 3), ddPCR/PCR with Sanger sequencing or immunohistochemistry staining (IHC). The detailed information of all patient characteristics is outlined in Fig. 1a and respective treatment regimens in Supplementary Table 1. A Venn diagram is shown in Fig. 1b, providing an overview of analyzed and overlapping LB probes and tissue samples across the cohort and the longitudinally followed cases and time points are shown in Supplementary Table 2. Only cases with at least 3 time points are shown.

### Liquid biopsy collection

CSF was collected from either the ventricles or the cisternal/ subarachnoid space at the time of tumor surgery (hereafter named as intra-operative), and by puncture of an Ommaya Reservoir or VP shunt (hereafter summarized as ventricular) or by lumbar puncture at clinically indicated time points in the post-operative period. CSF was stored at − 80 °C. EDTA blood tubes were processed within 60 min from the collection time and centrifuged for 10 min at 1000 g to separate the plasma from the solids. CSF samples from patients diagnosed with other pediatric brain tumors and plasma samples from patients with no tumor disease were included as controls. We pooled and aliquoted the plasma of 20 control patients. To avoid thawing and freezing cycles, all LB samples were aliquoted before the first freezing step at − 80 °C.

### DNA extraction from tumor tissue

Genomic DNA (gDNA) of frozen tumor tissue samples obtained from surgery were isolated with the ReliaPrepTM gDNA Tissue kit from Promega. The isolated gDNA was measured with a NanoDrop 1000 system from Thermo Scientific. For the analysis, a total amount of 10 ng gDNA was used.

### Cell free DNA (cfDNA) extraction from liquids

Prior to isolation, the thawed LB samples were centrifuged at 12.000 g for 15 min to remove cell debris. In total, 500 µl–1 ml of the LB sample was used for cfDNA isolation. The cfDNA extraction was performed using the Quick cfDNA/cfRNA Serum and Plasma kit from Zymo Research and processed according to the manufacture’s manual. In the final step, cfDNA was eluted with 20 µl nuclease-free water and stored immediately at − 80 °C.

### Pre-amplification PCR

A pre-amplification step for the cfDNA samples was performed using the Sso-AdvancedTM PreAmp Supermix from BioRad. We used the previously described pre-amplification primer pairs designs for the mutation H3F3A K27M and BRAF V600E [29] and purchased the oligo primer from Integrated DNA Technology (IDT). The pre-amplification reaction was mixed according to the instructor’s manual and performed in a BioRad C1000 Touch thermocycler at 95 °C for 3 min and 10 cycles of 95 °C for 15 s (denaturation) and 58 °C for 4 min (annealing and extension). The pre-amplification product was diluted 1:4 with nuclease-free water and stored at − 20 °C.

### Droplet digital PCR (ddPCR)

The QX200TM ddPCR system from BioRad was used and the rare event detection was performed according to manufactures manuals. In brief, the unique assay ID dHsaMDV2510510 for *the H3F3A K28M* mutation and dHsaMDV2010027 for the *BRAF V600E* mutation from BioRad was used to analyze the mutations in cfDNA of patient CSF/plasma samples. Prior to the analysis, a limit of detection curve was established by spiking either H3F3A K27M or BRAF V600E mutated cells with D425 medulloblastoma cells (as demonstrated in Supplementary Fig. 2 and 3). To each run, a sample with known positive H3F3A K27M or BRAF V600E mutation and a negative control (nuclease-free water) was included to determine the fluorescence thresholds (H3F3A K27M channel 1: 1000; channel 2: 1500; BRAF V600E channel 1: 1645; channel 2: 2000). To enrich the number of accepted droplets in total (approx. 3 × 10^4^–1 × 10^5^), we performed at least two repetitions for each CSF sample and five repetitions of each plasma sample. One run had to be clearly above the threshold to be counted as a successful positive signal. The results of ddPCR were analyzed with QuantasoftTM software, and the mutation of allele frequency in percent (MAF) [29] was calculated as follows = (positive mutation droplets/sum of positive MT and positive WT droplets) × 100.

### Radiology review

Radiology images were analyzed retrospectively. Magnetic resonance imaging (MRI) sequences were obtained at a 1.5 Tesla MRI scanner. MR imaging protocols encompassed at least T2-weighted sequences, fluid-attenuated inversion recovery (FLAIR) sequences and T1-weighted sequences with and without the intravenous application of gadolinium-based contrast media. All images were reviewed by the same board-certified neuro-radiologist (J.F.) employing the open-source segmentation software ITK-SNAP (version 3.6.0). A manual assessment of non-contrast-enhanced and contrast-enhanced tumor volume was performed on FLAIR MR sequences and T1-weighted contrast-enhanced MR sequences, respectively.

### Statistical analysis

Statistical analyses were performed with Microsoft Excel software, GraphPad Prism software Version 8 and IBM SPSS Statistics 27.

## Results

### Limit of detection of ddPCR system

To develop and test our LB monitoring method, we initially conducted a limit of detection experiment for H3F3A K27M and BRAF V600E mutation. We spiked the DNA of a medulloblastoma cell line (D425Med) with decreasing concentration of either H3F3A K27M or BRAF V600E mutated DNA and reached a limit of detection of 0.0025% for H3F3A K27M and 0.01% for BRAF V600E, respectively (Supplementary Fig. 2 and 3). For future analyses, we kept the same threshold settings for the channels.

### Patient characteristics and treatment

We analyzed 35 patients with a median age of 9.3 years (range 0–33, Fig. 1a–b, Supplementary Table 1,) 27 cases were classified as grade 3/4 glioma (HGG) tumors and 8 as grade 1/2 tumors (LGG). In 22 patients, H3F3A K27M was diagnosed, 12 had BRAF V600E mutation, and one was identified with both mutations. 23 patients had a biopsy, in eight patients, a partial resection or near total resection was performed, and in four patients, a complete resection was achieved. First-line chemotherapy was temozolomide in 23 cases, while seven received targeted therapy with trametinib/dabrafenib, vemurafenib, nivolumab/nimotuzumab or dendritic cell vaccination. Five patients were treated with carboplatin/vincristine. Two patients did not receive systemic therapy. All except eight received at least one course of radiation therapy and in twelve cases, re-irradiation therapy was conducted. The median overall survival of DMG H3K27M diagnosed patients was 22.18 months (Supplementary Fig. 4a). For BRAF V600E, we divided the cohort into LGG (WHO grade 1 and 2) and HGG (WHO grade 3 and 4) and reached a 5-year overall survival of 100% in the LGG group (*n* = 3), whereas the HGGs (*n* = 4) displayed a median survival of 93 months (Supplementary Fig. 4b). The BRAF V600E MAF detection in plasma samples showed no significant differences between WHO grades 1–4 (shown in Supplementary Fig. 4c). In total, 23 patients of the cohort had succumbed to their disease, while the remaining 12 patients were alive at the time of analysis.

### Validation of H3F3A K27M and BRAF V600E mutation in tumor tissue vs CSF and plasma

**Fig. 2:**
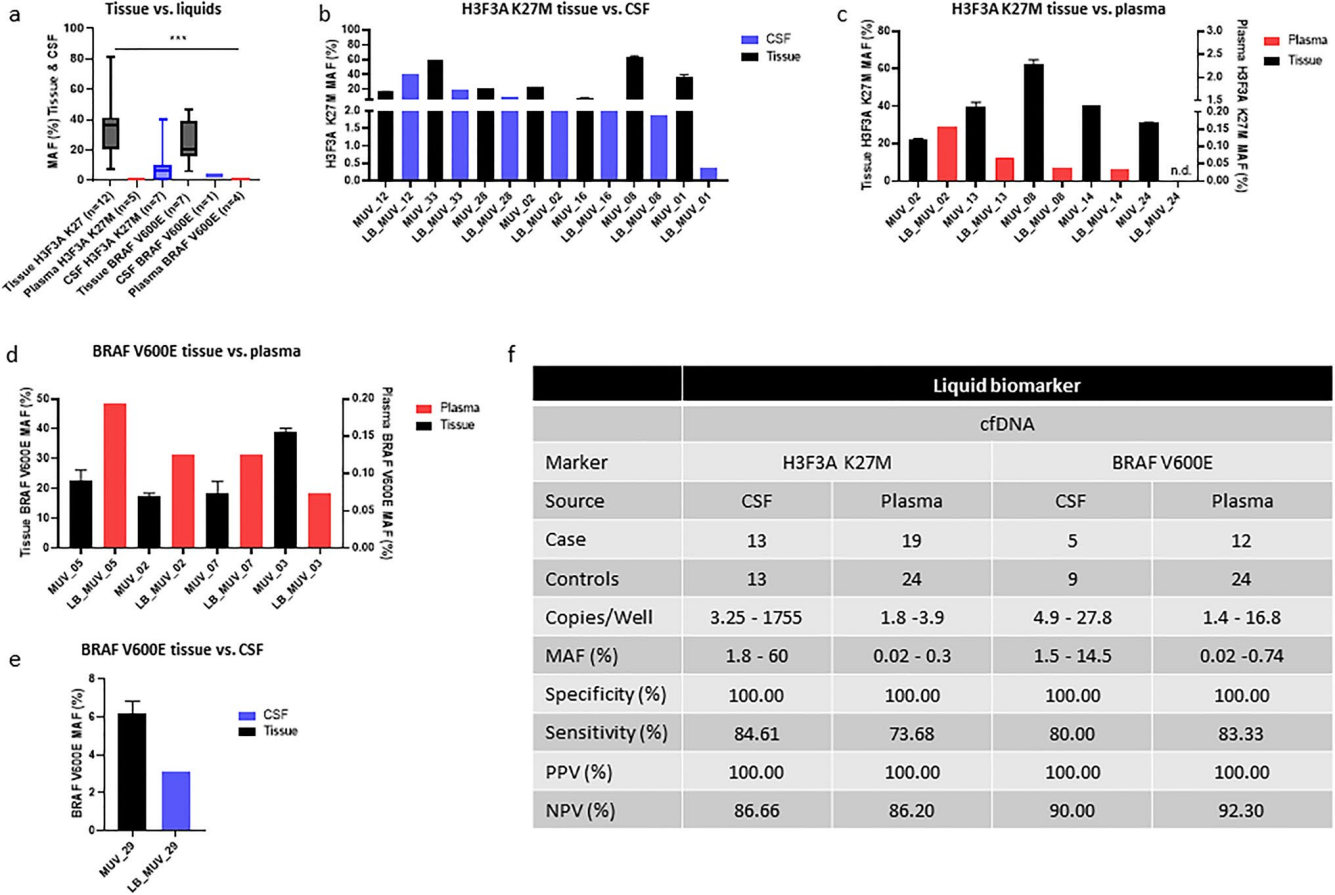
Quality assessement overview of liquid biomarkers: **a** mutation allele frequency (MAF) detection in different analytes, H3F3A K27M detection in **b** CSF vs tumor tissue, and **c**) plasma. BRAF V600E detection in **d** plasma and **e** CSF vs tumor tissue. Asterisks indicate significance (one-way Anova; ***P < 0.001 analyzed with GraphPad Prism), error bars indicate mean ± S.D.; *n.d.* not detected in the sample. **f** Specificity and sensitivity of liquid biomarkers in different analytes compared to non-tumor controls. Sensitivity, specificity and predictive values were calculated according to Trevethan R, 2017

First, we analyzed the tumor tissue of 20 patients in our cohort and isolated the genomic DNA (11 H3F3A K27M, 8 BRAF V600E and 1 H3F3A K27M/BRAF V600E double mutation) to validate our ddPCR method. All 20 tumor tissues displayed the known mutations with mean MAF of 34% in H3F3A K27M (ranging from 8 to 80%) and 25% in BRAF V600E (ranging from 4 to 40%), respectively (Fig. 1a). Subsequently, we screened our LB samples for the nearest time point to the surgery date where tumor was measured in MRI (CSF and plasma, if available, showed in Supplementary Table 3) and analyzed the matched LB MAF profile. In all CSF samples, we were able to detect H3F3A K27M with a mean value of 9% (ranging from 1.8 to 60%). In plasma samples, four of five patients showed the H3F3A K27M mutation with a mean value of 0.05% (range 0.02–0.3%) and all four patients had the BRAF V600E mutation with a mean of 0.1% (ranging 0.02–0.2%, Fig. 2b–d). For BRAF V600E, we had one matched CSF sample at diagnosis and detected a mean value of 3.12% (Fig. 2e). However, no other matched samples at diagnosis of BRAF V600E-positive cases were available. To validate the sensitivity of the detection system, we included CSF samples of other pediatric brain tumor entities (medulloblastoma *n* = 5, DMG/pons glioma H3F3A WT *n* = 3, pilocytic astrocytoma *n* = 2, HGNET-BCOR *n* = 1, ependymoma *n* = 1, LGG *n* = 1, GBM *IDH1 MT* *n* = 1,) and a serum pool of 20 non-tumor bearing pediatric patients.

**Fig. 3:**
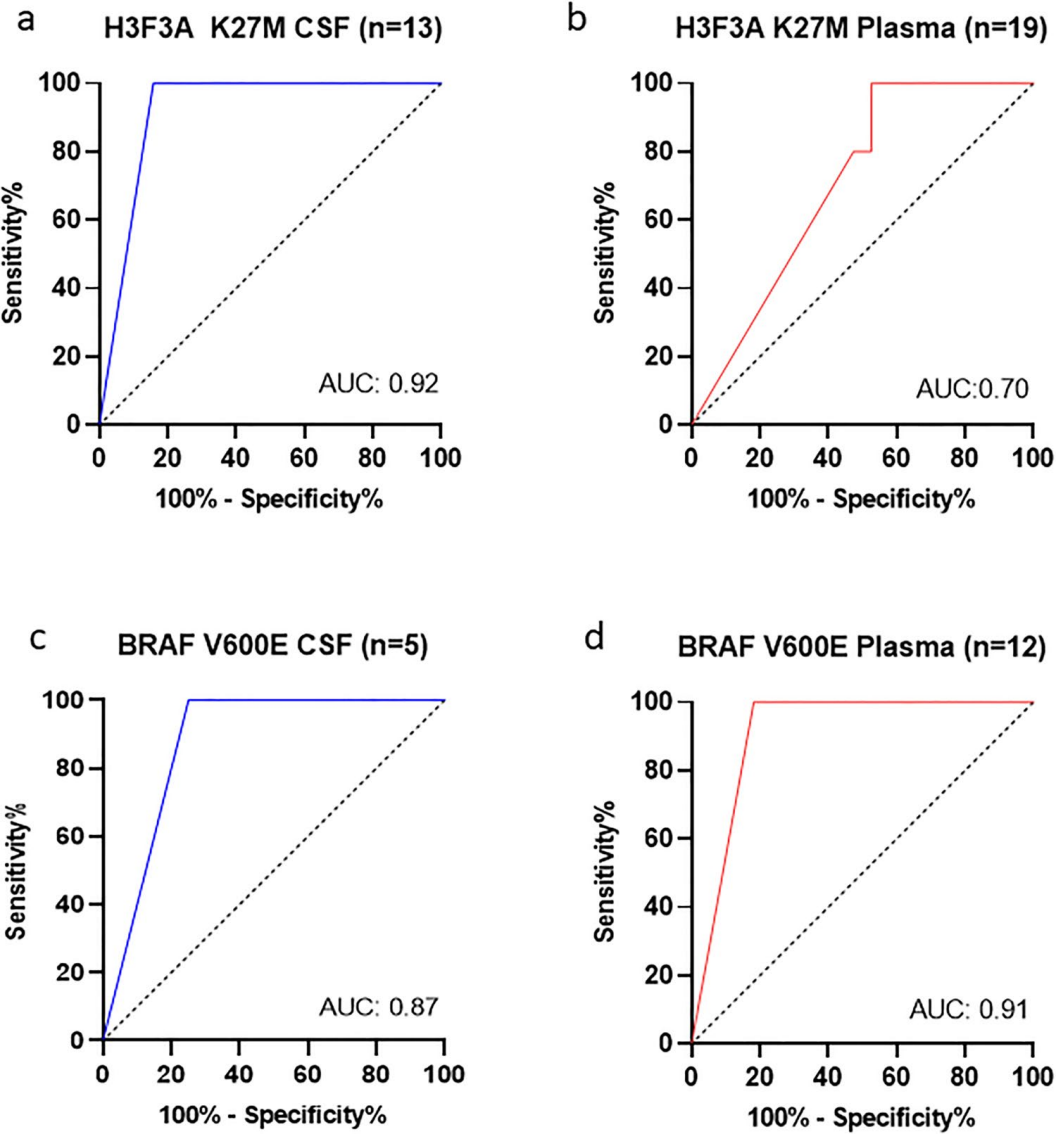
Receiver operation characteristics (ROC) curve of H3F3A K27M marker in **a** CSF (*n* = 13) and **b** plasma (*n* = 19). ROC curve of BRAF V600E marker in **c** CSF (*n* = 5) and d) plasma (*n* = 12). ROC curves were analyzed in IBM SPSS statistics 27 and mapped with GraphPad Prism

Moreover, we performed a quality check of our detection platform and screened the available CSF and plasma samples of our cohort (Fig. 2f). Including the previously mentioned matched biopsy samples, an additional five CSF (total *n* = 13) and 13 plasma samples (total *n* = 19) were available for H3F3A K27M (Supplementary Fig. 5a–b), and four more CSF samples (total *n* = 5) as well as 7 plasma samples for BRAF V600E detection (total *n* = 12) (Supplementary Fig. 5c–d). Based on these data, we calculated the predictive power as described by Trevethan in 2017 [37]. In the H3F3A K27M cohort, we reached a sensitivity of 84.61% for CSF and 73.68% for plasma and 100% specificity in both the sources. For the BRAF V600E cohort, we achieved a sensitivity of 83.33% and 100% specificity in the plasma group. For CSF, we found a sensitivity of 80% with 100% specificity (Fig. 2f). Furthermore, we performed receiver operation characteristics (ROC) curves to check the quality of the liquid biomarker of all LB samples and determined an area under the curve (AUC) of 0.92 in CSF and 0.70 in plasma for H3F3A K27M and 0.87 in CSF and 0.91 in plasma for BRAF V600E (Fig. 3a–d).

### LB detection rates differ between tumor and sampling site/time

**Fig. 4:**
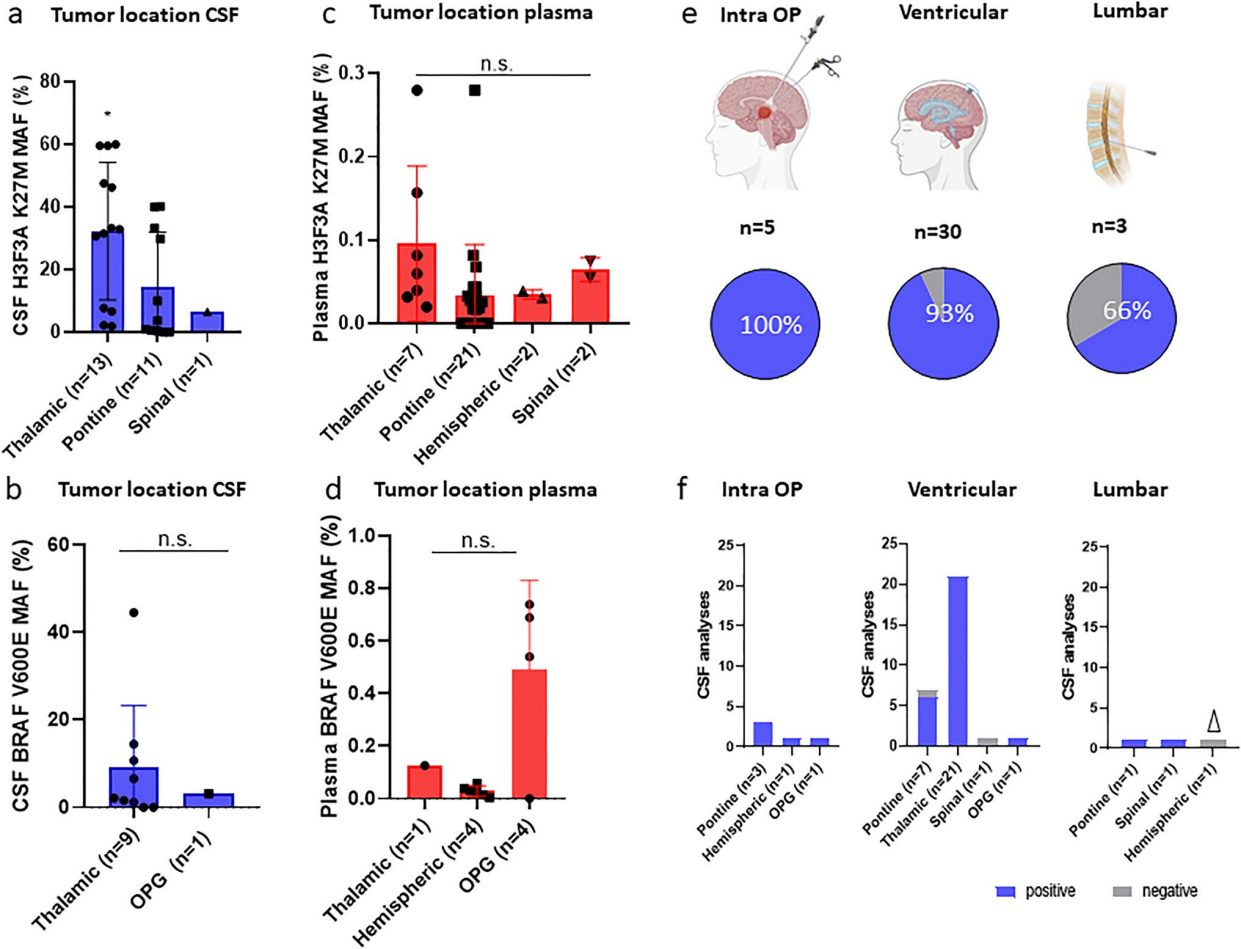
Tumor localization and LB detection. **a**–**d** Detection of H3F3A K27M and BRAF V600E classified to tumor location in CSF and plasma. **e** Pie chart of successful liquid biopsy according to puncture localization. **f** Detailed bar chart—tumors were grouped according to their localization and linked to successful LB detection. Asterisks indicate significance (students *t*-test; *P < 0.05, **P < 0.01 analyzed with GraphPad Prism), *n.s.* no significance, error bars indicate mean ± S.D. Parts of the figure were created with Biorender. OPG, optical pathway glioma. Δ indicated no tumor visible in MRI at LB sampling and ddPCR was negative for respective biomarker

Next, we correlated the primary tumor location (thalamic, pontine, hemispheric, optical pathway and spinal) to the successful detection of the biomarkers. Except for a higher H3F3A K27M mutation detection in CSF of patients with a thalamic location (MAF range from 1.8 to 60%, *p < 0.05), we did not observe a significant difference among tumor locations as shown in Fig. 4a–b. There was a tendency though for a better detection result in thalamic tumors followed by pontine and spinal gliomas. However, this tendency might have been confounded by the fact that most of the available CSF samples were from cases with tumors located in the thalamic area. Hence, we can not exclude a selection bias. Interestingly, the same trend in thalamic tumors was observed for plasma in the H3F3A K27M group (Fig. 4c). In the BRAF V600E sample collection, the optical pathways glioma (OPG) displayed the highest detection rate (Fig. 4d). However, there was no significant difference between the three brain locations.

Consequently, we analyzed the potential impact of the sampling time and site and grouped our CSF samples into intra-operatively collected CSF, and post-operatively collected CSF from ventricular or lumbar puncture sites. The most sensitive sampling method regardless of the tumor location and sampling site was the intra-operatively collected CSF (100%, cisternal/subarachnoidal *n* = 3, ventricular *n* = 1, biopsy canal *n* = 1) followed by ventricular CSF (93%, *n* = 30) and the lumbar puncture site (66%, *n* = 3) as shown in Fig. 4e. Subsequently, we analyzed the correlation between tumor location and puncture site/time (Fig. 4f). In the pontine group (*n* = 11), we had three intra-operative CSF samples with 100% (3/3) detection, the ventricular site showed a positive signal in six of seven samples (85%) and in the single case obtained by lumbar puncture (100%). One CSF sample of an OPG case was obtained intra-operatively and showed a positive signal (*n* = 1). In the thalamic group (*n* = 21), we could detect the underlying alteration in 100% of ventricular CSF. In this group, no other detection sites were available. With respect to hemispheric tumors, mutation detection by LB was positive in one (*n* = 1) intra-operative sample but not in one lumbar puncture sample, notably, at time of LB sampling, this case had no visible tumor in the MRI (*n* = 1). The spinal cohort included one patient with CNS metastasis at several sites. In this case, analysis was successful (100%), whereas the ventricular puncture did not show mutation detection. In summary, we identify a potential impact of the CSF sampling site in relation to tumor localization which is of potential relevance for LB detection in brain tumors.

### Radiological features

Next, we correlated LB parameters to radiological findings. We compared the contrast-enhancing and non-contrast-enhancing tumor volume to matched LB measurements in plasma (*n* = 19) and CSF (*n* = 9). Within our limited cohort, we did not detect an overall correlation between tumor volume and MAF in either plasma or CSF (Supplementary Fig. 6). Importantly, all patients with leptomeningeal tumor dissemination showed detectable ctDNA in CSF pointing towards a particular potential for detection and tumor surveillance in this patient group (Supplementary Fig. 7a). In contrast, positive LB detection in plasma was not associated with leptomeningeal metastasis, and in both liquids, the presence of tumor necrosis had no impact on LB (Supplementary Fig. 7b–d).

### Longitudinal follow-up in plasma and CSF

**Fig. 5:**
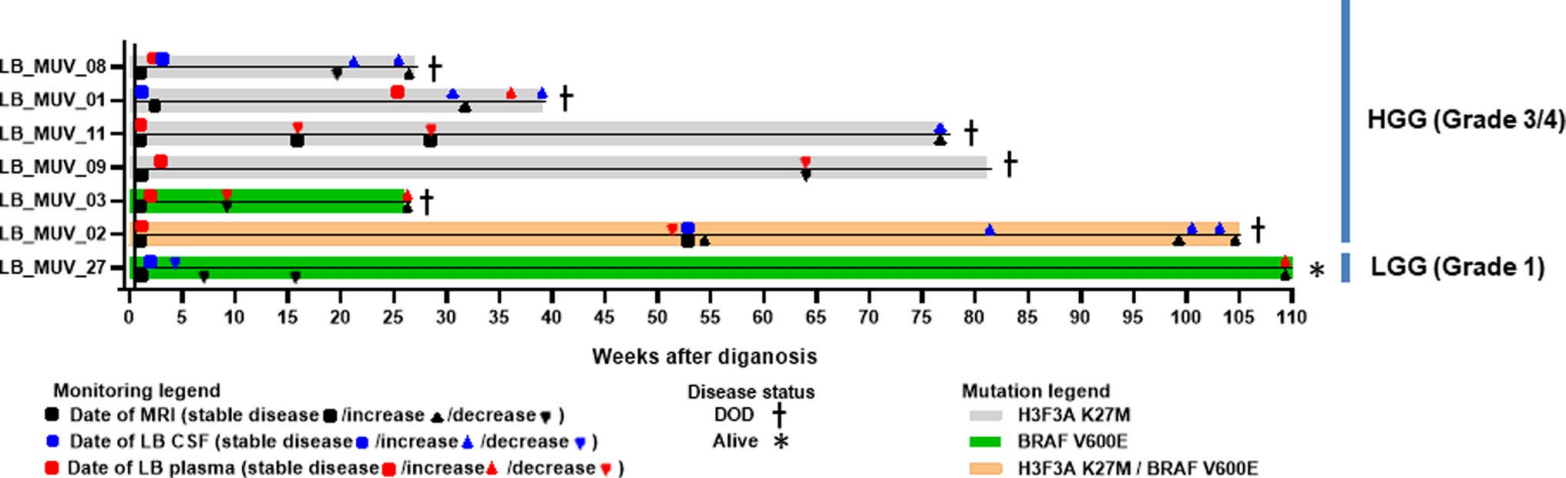
Swimmer plot of longitudinal LB monitoring of 7 cases including all MRI and LB time points. The first squares in the timeline represent the initial MRI and LB time point of each patient; more squares in the timeline depict a stable disease; triangles demonstrate an increase or decrease in in tumor growth and LB mutation detection. The BRAF V600E patients represent one LGG WHO grade 1 (LB_MUV_27 diagnosed as OPG) and one HGG WHO grade 4 (LB_MUV_03 diagnosed as gliosarcoma)

**Fig. 6:**
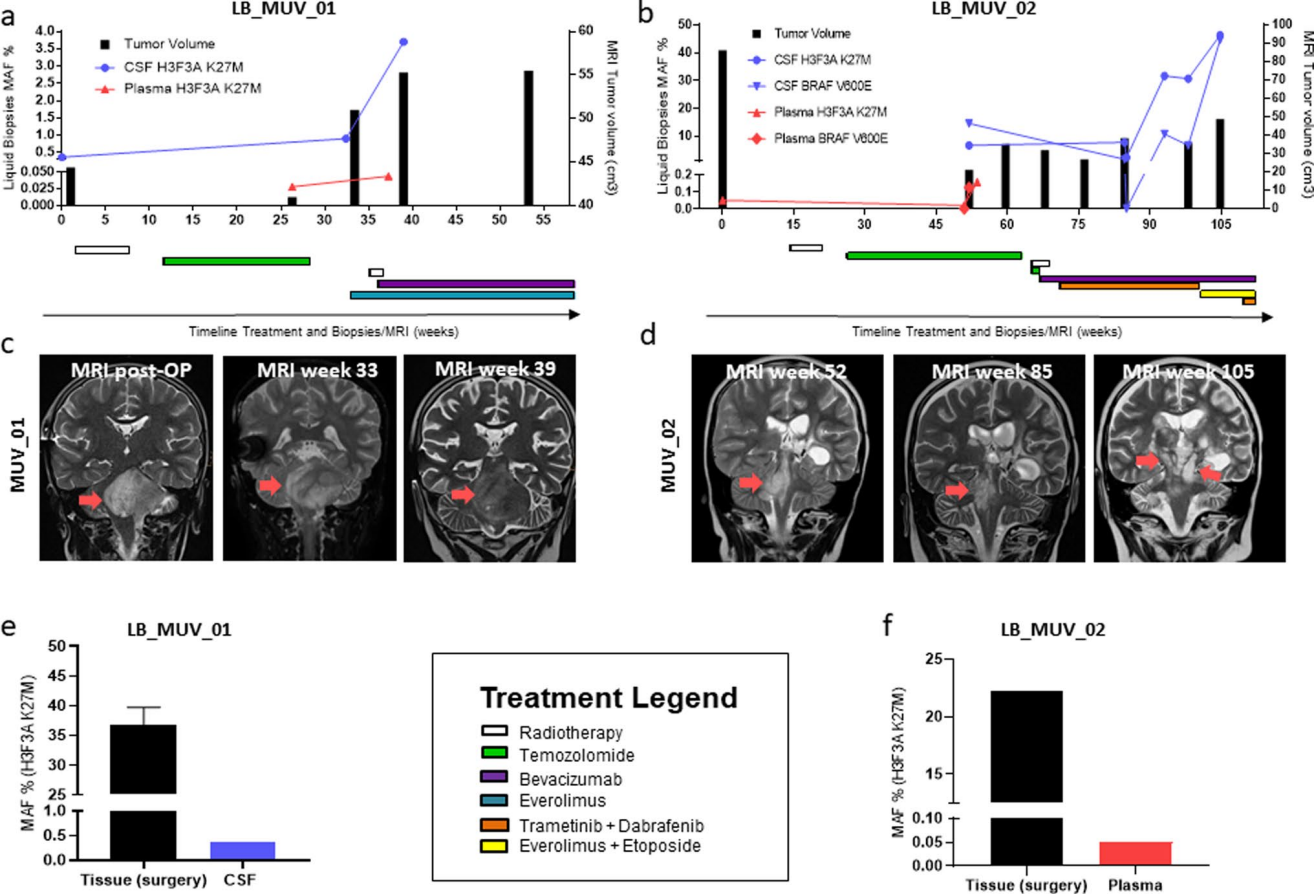
Longitudinal monitoring of 2 cases. Treatment history including MAF of LB and tumor volumes obtained from MRI (cm^2^) of **a** LB_MUV_01 and **b** LB_MUV_02. Matched MRI images (coronal T2-weighted MRI) to LB MAF detection for **c** LB_MUV_01 and **d** LB_MUV_02. Red arrow marks the tumor spread. Initial MAF of H3F3A K27M in tissue vs initial pre-surgery LB samples: **e** CSF pre-surgery and **f** plasma

Finally, we analyzed the longitudinal monitoring opportunities of our biomarkers in CSF and/or plasma of seven patients. Four patients harbored a H3F3A K27M mutation (LB_MUV_01, LB_MUV_08, LB_MUV_09 and LB_MUV_11), two a BRAF V600E mutation (LB_MUV_03 and LB_MUV_27) and one case was characterized by both mutations (LB_MUV_02). Figure 5 depicts a swimmer plot, indicating the longitudinal monitoring time points, and showing the increasing or decreasing levels of detected biomarkers in CSF or plasma and the concurrent MRI results. We could demonstrate the increase of mutational DNA fraction of H3F3A K27M in the CSF and plasma in patients with a tumor progression in the MR images (Fig. 6a–d, Supplementary Fig. 8a–b, Supplementary Fig. 9) and partially decreased levels for BRAF V600E mutation during targeted drug treatment with trametinib and dabrafenib (Fig. 6b, Supplementary Fig. 10). However, in LB_MUV_19, a slight increase of H3F3A K27M was detected in the last obtained plasma, while the tumor mass in the last MRI was shrinking (Supplementary Fig. 9c). It is worth noting that in three patients (LB_MUV_01, LB_MUV_02 and LB_MUV_08), we could compare tumor tissue and liquids obtained on the day of surgery and detected positive results in our detection platform in all available liquids as shown in Fig. 6e–f and Supplementary Fig. 8c. These results support the strong significance of ddPCR-based LB detection and its clinical relevance for diagnosis and longitudinal treatment monitoring.

## Discussion

LBs of CSF and plasma in brain tumor patients offer novel opportunities for more rapid and less invasive diagnosis compared to surgical biopsies as well as longitudinal disease monitoring with the potential to revolutionize therapeutic management. However, there are still many limitations and obstacles in the generation and interpretation of LB results. Among these, sample generation, minimum quantities and processing are the main challenges [1, 5, 20, 25, 28, 29, 32, 40]. Within our cohort, we extracted cfDNA from a minimum of 500 µl for CSF and 1 ml of plasma and aimed to assess the extreme sensitive ddPCR-based LB detection in LGG and HGG. Moreover, clinical parameters such as therapy, tumor composition and relation of CSF site to tumor site may impact the LB result [5, 25, 29]. For example, previous studies showed an increase of mutant cfDNA levels in LB upon irradiation of tumors [5, 21, 29]. However, the impact of these factors is still not clear which limits the interpretation of LB results in the clinical setting. During the last decade, we and other groups investigated the molecular biology of these DMG H3F3A K27M tumors and identified several well-known oncogenic driver alterations and pathways to be dysregulated in H3F3A K27M glioma [4, 11, 12, 16, 19, 38]. The described pathways and mutations include major genes of the cell and DNA repair pathway (*TP53*, *PPM1D*, *ATM*, and *ATRX*) and the receptor tyrosine kinase pathway (*ACVR1*, *FGFR1*, *PIK3CA*, *PIK3R1*, and *BRAF*) [11, 12, 30]. These studies also demonstrated the heterogeneity of this tumor class. In some cases, a co-occurrence of H3F3A K27M and BRAF V600E mutation in midline gliomas grade 1 and ganglioglioma was reported [27]. The occurrence of this double mutation is associated with a better median survival of the patients compared to H3F3A K27M alone.

Recently, the clinical feasibility of targeting H3F3A K27M in serially collected plasma and CSF samples obtained from patients diagnosed with a DMG H3K27M alteration was investigated [5, 29]. In this study, the authors examined the novel mono-therapeutic agent ONC201 and demonstrated the ability to use LB samples to monitor treatment efficacy. In our longitudinally monitored patients with BRAF V600E mutation, we observed similar results and detected reduced values in both fluids during the targeted therapy with trametinib and dabrafenib. Notably, in our cohort, BRAF V600E detection in the plasma showed the best quality characteristics according to sensitivity calculations and ROC curves. Therefore, plasma from BRAF V600E mutated patients could be increasingly used to monitor targeted therapies. However, the study cohort was small (*n* = 12) and a batch bias cannot be excluded. We, therefore, suggest an extended analysis in a larger prospective cohort. The observed significance should also be validated. The H3F3A K27M study group demonstrated the best results in the CSF and a correlation between the positive LB detection and leptomeningeal metastases were determined. Here, we correlate the LB results to radiological findings such as necrosis to interrogate this question; however, within our small cohort, we did not detect a correlation and can thus neither confirm nor exclude such an effect.

In 2021, Eibl et al. [10] showed that CSF puncture from the intracranial region outperformed lumbar puncture and increased the performance of positive LB analysis. In our study, we were able to confirm this observation and even expand and support it with more samples and additional puncture sites. All CSF samples obtained intra-operatively exhibited a positive LB result. This observation supports the potential for liquid biopsy for analysis of CSF samples potentially obtained prior to tumor surgery upon shunt/extra ventricular drain implantation as well as within the operation time upon longer surgical procedures. The ventricular CSF, taken via Ommaya Reservoir or shunt puncture, showed a positive signal in 93%, and 66% in lumbar punctures. Moreover, we were able to demonstrate that the relation of tumor localization and to the CSF sampling site significantly influence the result of LB. In LB_MUV_16, the tumor was situated in the spine, and the lumbar puncture yielded positive findings. In contrast, the ventricular puncture failed to mirror these results, and the LB returned negative results. These observations should be taken into account in the decision-making process regarding the location of the puncture for successful longitudinal monitoring.

## Conclusion

In conclusion, we present for the first time that BRAF V600E mutation detection in longitudinal LB samples may serve as monitoring biomarker and demonstrate an association between positive ddPCR results and leptomeningeal dissemination in the DMG H3K27 group. Importantly, the number of cases is small and the results need to be validated in a larger cohort. Another important aspect of this study is the clinical applicability and significance of LB samples for tumor monitoring in DMG H3K27 patients when surgery would be associated with high risk. Finally, and most importantly, the puncture site has a significant impact on the success of LB analysis.

## Supplementary Information

Below is the link to the electronic supplementary material.

Supplementary file1 (PDF 1662 KB)

## Data Availability

The datasets supporting the conclusions of this article are included within the article and its additional files. Any additional data are available on request from the corresponding author, due to privacy restrictions.
