# Supplementary material for: Detection of H3F3A K27M or BRAF V600E in liquid biopsies of brain tumor patients as diagnostic and monitoring biomarker: impact of tumor localization and sampling method
Source: Acta Neuropathol. 2025 Jan 3;149(1):5. doi: 10.1007/s00401-024-02842-7 (PMC11698890; doi:10.1007/s00401-024-02842-7)
Supplement: Supplementary file 1 — Supplementary file1 (PDF 1662 KB) [file 401_2024_2842_MOESM1_ESM.pdf]

Supplementary Tables and Figures

Madlener et al.

Supplementary Table 1: Patient cohort

Case	Age (years)	Diagnosis	WHO Grade	Detection of molecuar alteration	Surgery	1st line CT	1st line RTX	2nd line RTX	intrathecal CTx	OS (months)	Status
LB_MUV_1	6	DMG H3K27M	4	IHC	Biopsy	Temozolomide	yes, local	yes	no	14	DOD
LB_MUV_2	8	DMG H3K27M, BRAF V600E	4	OCCRA	PR	Temozolomide	yes, local	yes	no	25	DOD
LB_MUV_3	12	Gliosarcoma, BRAF V600E	4	PCR + Sanger	PR/NTR	Temozolomide, Dabrafenib + Trametinib	yes, local	no	no	95	DOD
LB_MUV_4	10	DMG	4	IHC	Biopsy	Temozolomide	yes, local	no	no	19	DOD
LB_MUV_5	12	aPXA, BRAF V600E	2	PCR RGQ PCR Kit	PR	Vemurafenib	yes, local	no	no	118	alive
LB_MUV_6	17	Glioblastoma	4	ddPCR	CR	Temozolomide, dendritic cell vaccination	yes, local	yes	no	155	alive
LB_MUV_7	17	PXA, BRAF V600E	2	ddPCR	CR	no	no	no	no	103	alive
LB_MUV_8	9	DMG H3K27M	4	IHC	PR	Temozolomide	yes, local	no	no	7	DOD
LB_MUV_9	12	DMG H3K27M	4	IHC	Biopsy	Temozolomide	yes, local	yes	no	21	DOD
LB_MUV_10	15	DMG H3K27M	3	OCCRA	CR	Temozolomide	yes, local	no	no	139	alive
LB_MUV_11	4	DMG H3K27M	4	OCCA v3	PR	Temozolomide	yes, local	yes	no	50	DOD
LB_MUV_12	6	DMG H3K27M	4	IHC	Biopsy	Temozolomide	yes, local	no	no	21	DOD
LB_MUV_13	4	DMG H3K27M	4	IHC.	Biopsy	Temozolomide	yes, local	no	no	8	DOD
LB_MUV_14	14	DMG H3K27M	4	IHC	Biopsy	Temozolomide	yes, local	no	no	13	DOD
LB_MUV_15	3	DMG H3K27M	4	OCCA v3	Biopsy	Temozolomide	yes, local	yes	no	12	DOD
LB_MUV_16	14	DMG H3K27M	4	OCCRA	PR	Temozolomide	yes, local	yes	yes	30	DOD
LB_MUV_17	8	DMG H3K27M	4	IHC	Biopsy	dendritic cell vaccine	yes, local	no	no	10	DOD
LB_MUV_18	5	DMG H3K27M	4	IHC	Biopsy	Temozolomide	yes, local	no	no	11	DOD
LB_MUV_19	8	DMG H3K27M	4	IHC	Biopsy	Temozolomide	yes, local	yes	no	16	DOD
LB_MUV_20	11	DMG H3K27M	4	OCCRA	Biopsy	Temozolomide	yes, local	yes	no	19	DOD
LB_MUV_21	13	PXA, BRAF V600E	3	OCCRA	PR	Dabrafenib + Trametinib	yes, local	no	no	30	DOD
LB_MUV_22	8	DMG H3K27M	4	TSO500	Biopsy	Temozolomide	yes lokal	no	no	10	DOD
LB_MUV_23	33	DMG H3K27M	4	TSO500	PR	Temozolomide	yes, local	yes	no	14	DOD
LB_MUV_24	10	DMG H3K27M	4	IHC	Biopsy	Temozolomide	yes, local	yes	no	18	DOD
LB_MUV_25	18	DMG H3K27M	4	TSO500	Biopsy	Nivolumab, Nimotuzumab	yes, local	yes	no	18	DOD
LB_MUV_26	4	DMG H3K27M	4	IHC	Biopsy	Temozolomide	yes, local	no	no	6	DOD
LB_MUV_27	0	OPG, BRAF V600E	1	OCCRA	Biopsy	Dabrafenib + Trametinib	no	no	no	22	alive
LB_MUV_28	2	DMG H3K27M	4	TSO500	Biopsy	Temozolomide	yes, local	no	no	13	DOD
LB_MUV_29	16	PXA, BRAF V600E	3	TSO500	CR	no	no	no	no	11	alive
LB_MUV_30	13	BRAF V600E	1	IHC	Biopsy	Carboplatin, Vincristine	no	no	no	75	alive
LB_MUV_31	6	BRAF V600E	1	OCCA v3	Biopsy	Carboplatin, Vincristine	no	no	no	62	alive
LB_MUV_32	5	OPG, BRAF V600E	1	TSO500	Biopsy	Carboplatin, Vincristine	no	no	no	63	alive
LB_MUV_33	12	DMG H3K27M	4	IHC	Biopsy	Temozolomide	yes, local	no	no	1	alive
LB_MUV_34	2	OPG, BRAF V600E	1	OCCRA	Biopsy	Carboplatin, Vincristine	no	no	no	47	alive
LB_MUV_35	4	OPG, BRAF V600E	1	TSO500	Biopsy	Carboplatin, Vincristine	no	no	no	83	alive

Table legend: DMG, diffuse midline glioma; aPXA, anaplastic pleomorphic xanthoastrocytoma; PXA, pleomorphic xanthoastrozytoma; PA, pilocytic astrozytoma; OPG, optical pathway glioma; PR, partial resection; CR, complete resection; NTR, near total resection; OS, overall survival; RTX, radio therapy; CT, chemotherapy; DOD, death of disease.; IHC, immunohistochemistry; OCCRA, Oncomine Childhood Cancer Research Assay; OCCA V3, Oncomine Comprehensive Cancer Assay Version 3; TSO500, TruSight Oncology 500; ddPCR droplet digital PCR.

Supplementary Table 2: Detailed information of longitudinally monitored cases

Cases	Source	Marker	Longitudinal monitoring (time points)	Sampling sites CSF
LB_MUV_01	CSF	H3F3A K27M	3	Ventricular
LB_MUV_01	Plasma	H3F3A K27M	2	n.a.
LB_MUV_02	CSF	H3F3A K27M	6	Ventricular
LB_MUV_02	Plasma	H3F3A K27M	3	n.a.
LB_MUV_11	Plasma	H3F3A K27M	3	n.a.
LB_MUV_02	CSF	BRAF V600E	6	Ventricular
LB_MUV_02	Plasma	BRAF V600E	3	n.a.
LB_MUV_03	Plasma	BRAF V600E	3	n.a.
LB_MUV_09	Plasma	BRAF V600E	3	n.a.
LB_MUV_27	CSF	BRAF V600E	2	Ventricular
LB_MUV_27	Plasma	BRAF V600E	1	n.a.

n.a. not available

Supplementary Table 3: Detailed information on LB collection according to positive MRI, tissue measurements and surgical status

Case	Alteration	Source	preOP	postOP	Treatment (yes/no)	MRI positive (yes/no)
LB_MUV_01	H3F3A K27M	CSF	X		no	yes
LB_MUV_02	H3F3A K27M	CSF		X	yes	yes
LB_MUV_08	H3F3A K27M	CSF		X	no	yes
LB_MUV_12	H3F3A K27M	CSF	X		yes	yes
LB_MUV_16	H3F3A K27M	CSF	X		yes	yes
LB_MUV_26	H3F3A K27M	CSF		X	yes	yes
LB_MUV_33	H3F3A K27M	CSF	X		no	yes
LB_MUV_02	H3F3A K27M	Plasma	X		no	yes
LB_MUV_08	H3F3A K27M	Plasma		X	no	yes
LB_MUV_14	H3F3A K27M	Plasma		X	yes	yes
LB_MUV_24	H3F3A K27M	Plasma	X		no	yes
LB_MUV_27	BRAF V600E	CSF	X		no	yes
LB_MUV_02	BRAF V600E	Plasma	X		no	yes
LB_MUV_03	BRAF V600E	Plasma	X		no	yes
LB_MUV_05	BRAF V600E	Plasma		X	yes	yes
LB_MUV_07	BRAF V600E	Plasma	X		no	yes

Supplementary Figure 1: Graphical abstract of the study design

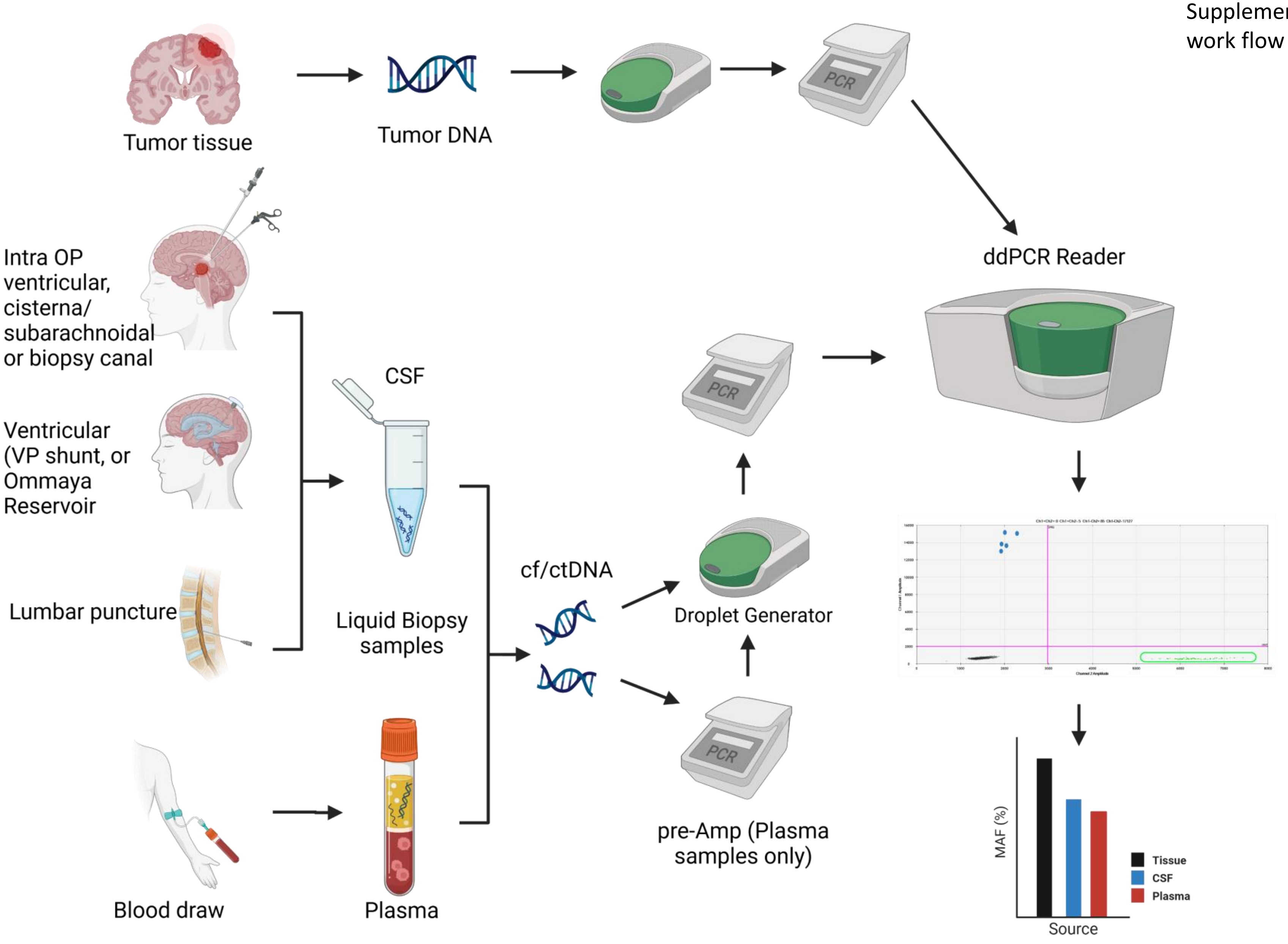

Supplementary Figure 1: Graphical illustration of study work flow and design. Figure was created with Biorender

Supplementary Figure 2: Limit of H3F3A K27M detection - ddPCR

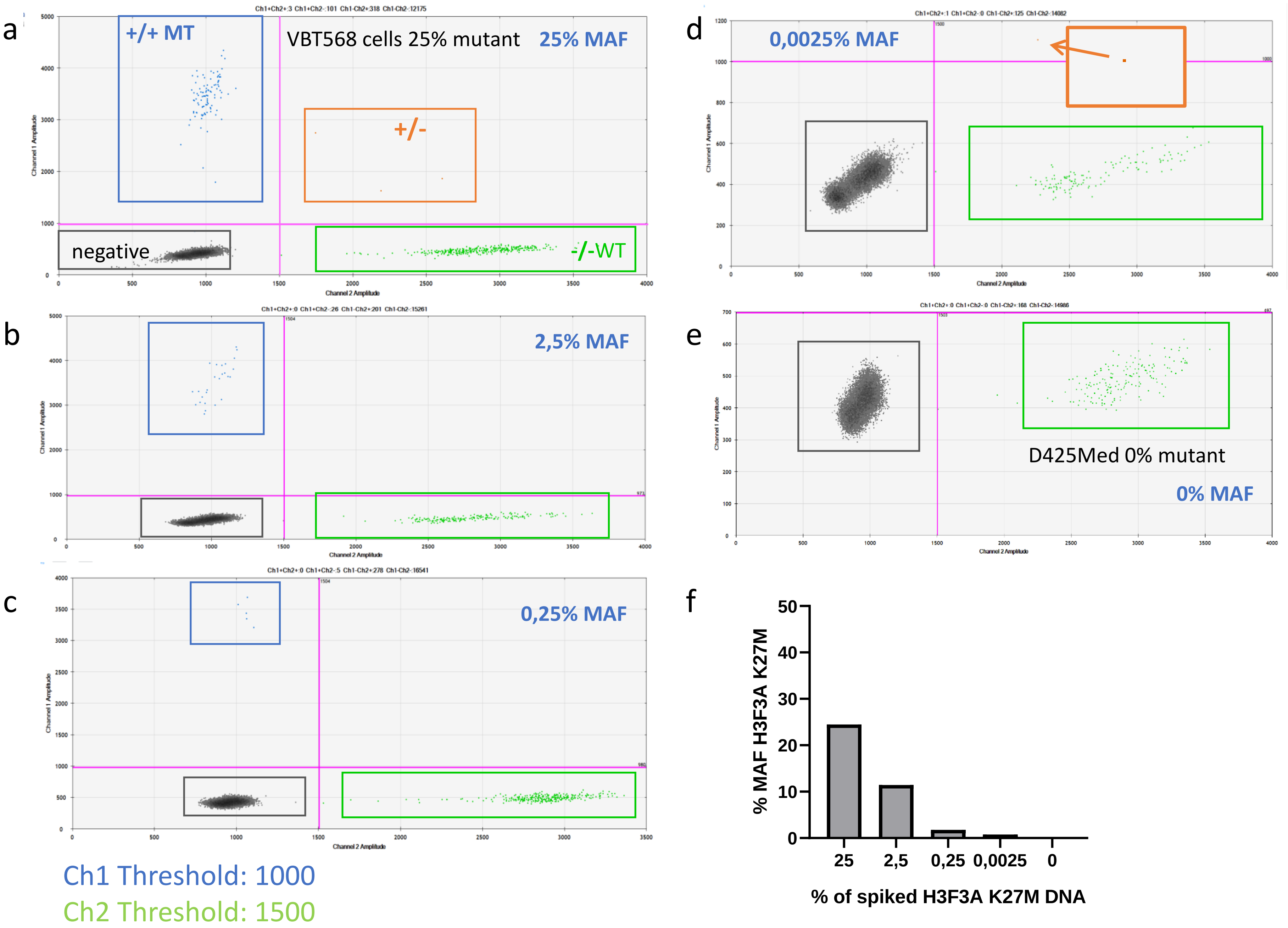

Supplementary Figure 2: H3F3A K27M expressing cells were spiked with D425Med cells to generate the limit of detection for H3F3A K27M. Serial dilutions were performed and scatter blots with detected droplets are represented in Figure a-e) and combined results are shown in Figure f) as bar graph. The threshold was set to 1000 for channel 1 (blue) and 1500 to channel 2 (green).

Supplementary Figure 3: Limit of BRAF V600E detection - ddPCR

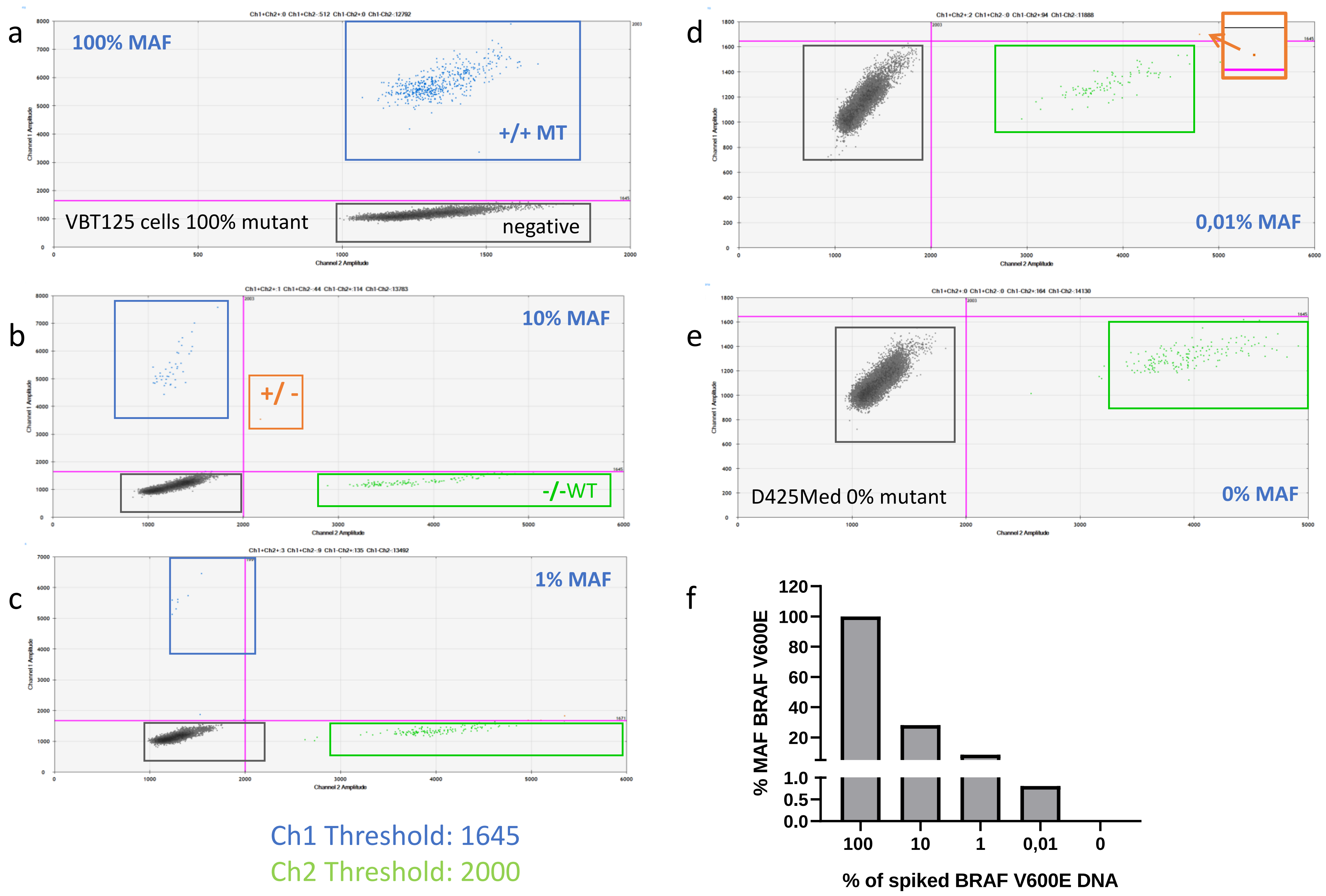

Supplementary Figure 3: BRAF V600E expressing cells were spiked with D425Med cells to generate the limit of detection for BRAF V600E. Serial dilutions were performed and scatter blots with detected droplets are represented in Figure a-e) and combined results are shown in Figure f) as bar graph. The threshold was set to 1645 for channel 1 (blue) and 2000 to channel 2 (green).

Supplementary Figure 4: Survival curve of patient cohort

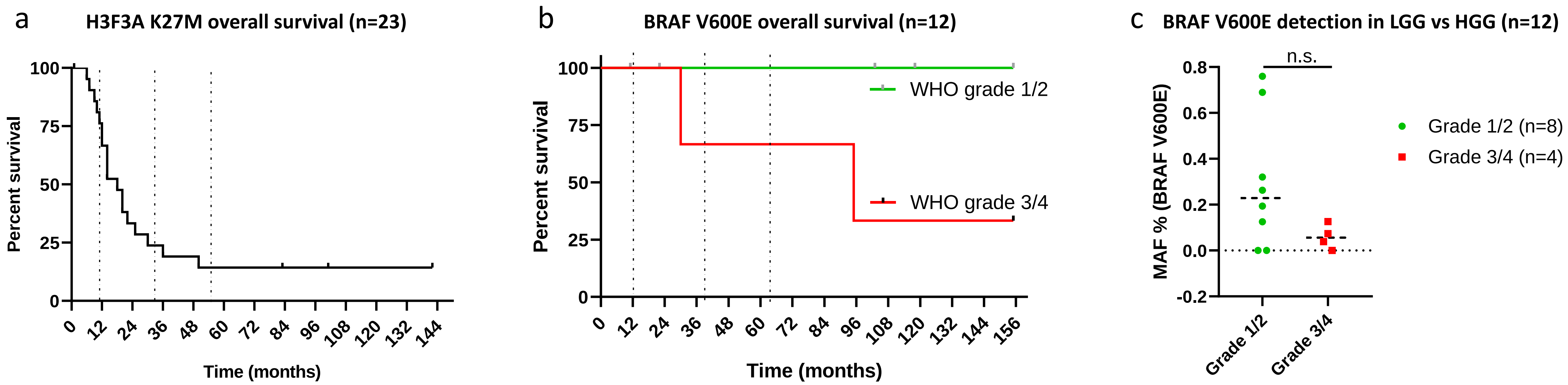

Supplementary Figure 4: a) represents the included H3F3A K27 cases, b) the Kaplan Meier Curve for BRAF V600E separated in WHO Grade 1/2 and 3/4 , c) MAF of BRAF V600E cohort separated into Grade 1/2 and Grade 3/4, individual values and median are indicated

Supplementary Figure 5: H3F3A K27M and BRAF V600E at later time points

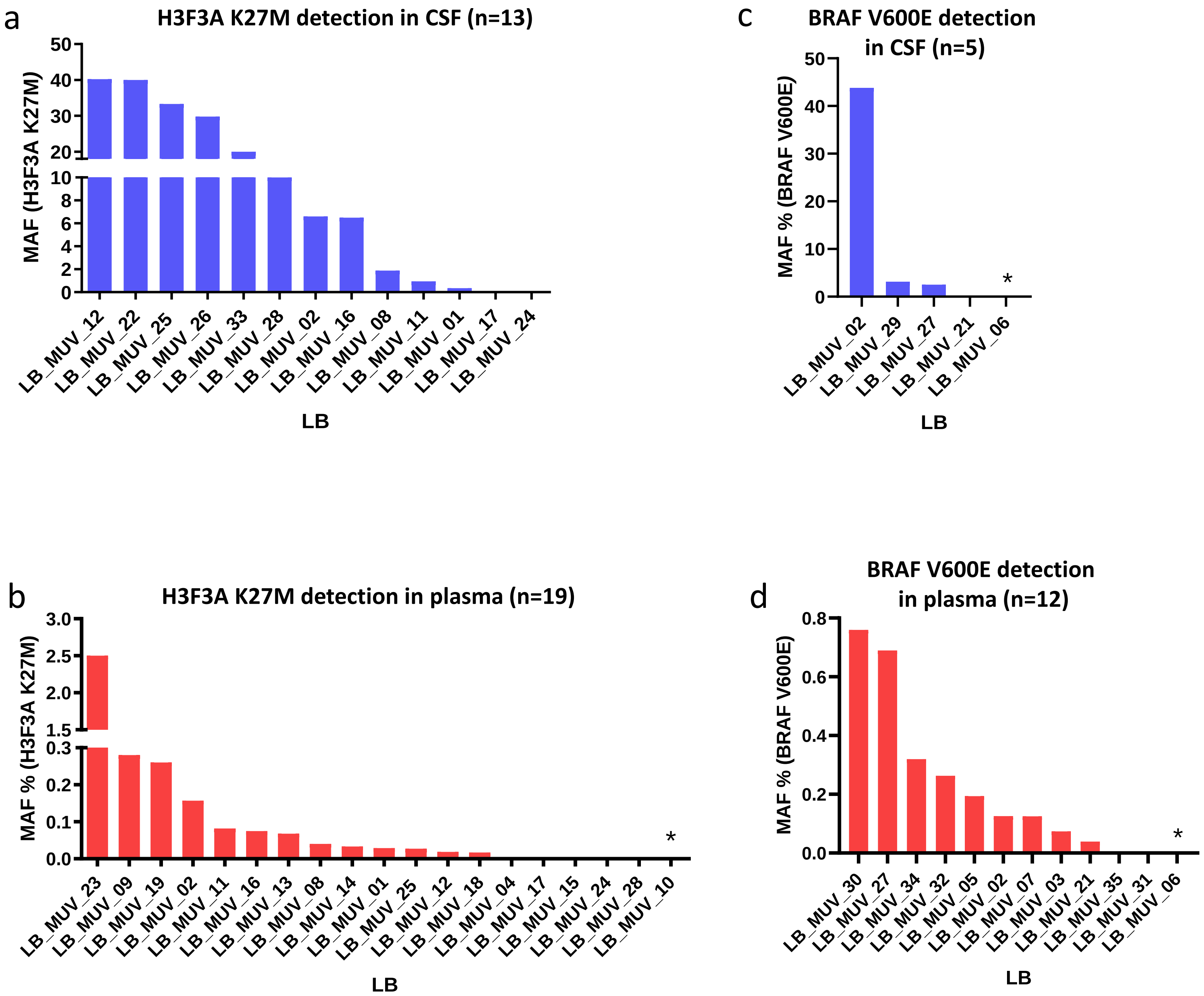

Supplementary Figure 5: Mutation allele frequency (MAF) detection of H3F3A K27M in a) CSF (n=13) and b) plasma samples (n=19). BRAF V600E MAF detection in c) CSF (n=5) and d) plasma (n=12). Graphs were generated with GraphPad Prism. Asterisks indicated no tumor visible in MRI and ddPCR was negative for respective biomarker

Supplementary Figure 6: Correlation liquid biopsies vs tumor size

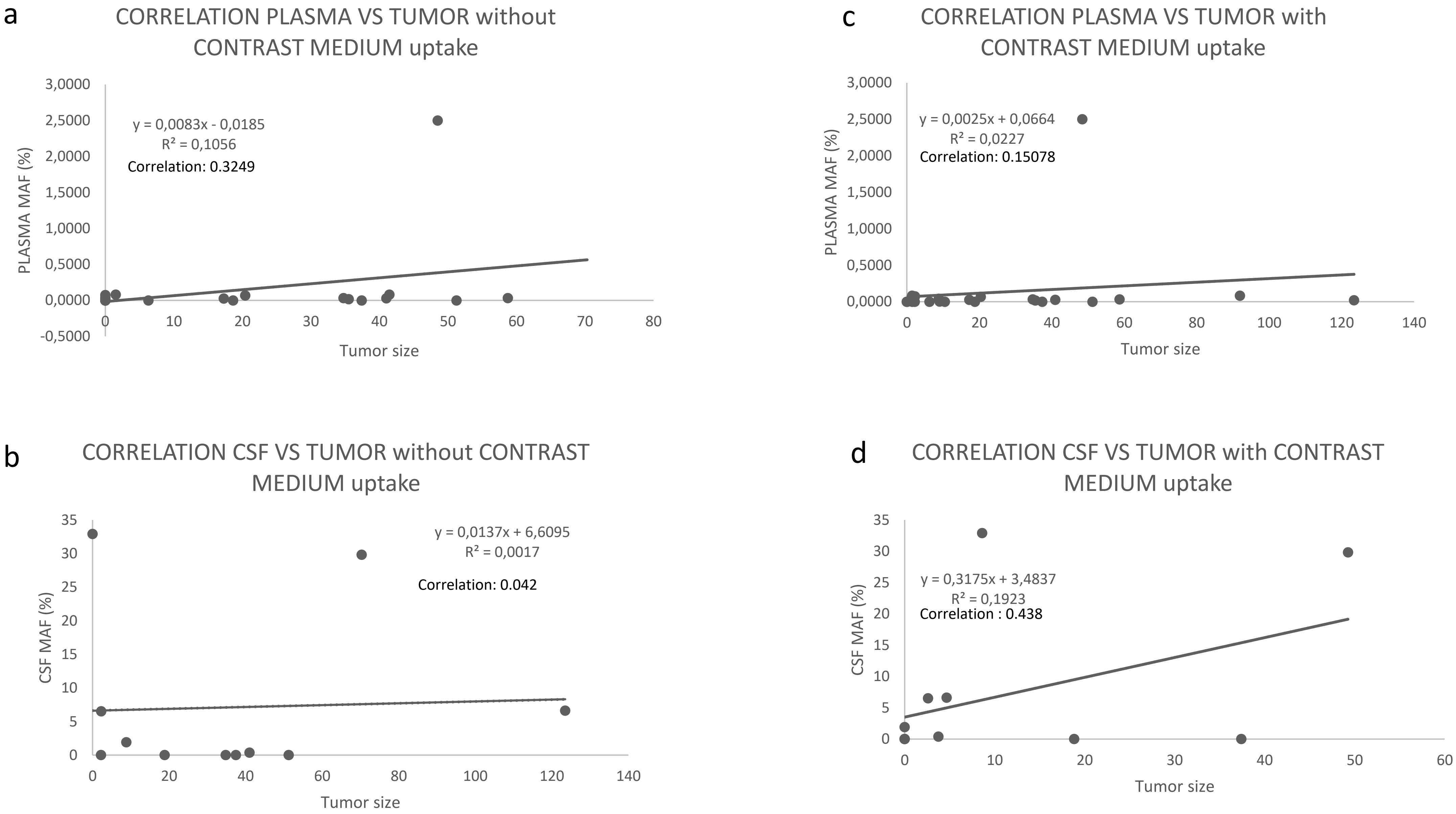

Supplementary Figure 6: Correlation plots: between a) plasma or b) CSF vs tumor without contrast medium, c) plasma or d) CSF vs tumor with contrast medium uptake. The plots were generated with IBM SPSS Statistics 27 software.

Supplementary Figure 7: Chi-Square tables

Supplementary Figure 7: Cross tabulation with Chi-square test. a) LB-CSF and leptomeningeal metastais, b) LB-plasma and leptomeningeal metastasis. c) CSF and necrosis, d) plasma and necrosis. Significnat p values are highlighted in red. Graphs were created with IBM SPSS Statistics 27 software.

a CSF and leptomeningeal metastasis

Leptomeningeal meatstasis

		Yes	No	Total
**CSF**	**Negative**	**0**	**29**	**19**
	**Positive**	**4**	**12**	**16**
**Total**		**4**	**31**	**35**

	Value	df	Asymptotic Significance (2-sided)
Pearson Chi-Square	5.363	1	0.021
Likelihood Ratio	6.882	1	0.009
N of Valid Cases	35		

p= 0.021 (significance between positiv LB in CSF and leptomeningeal metastasís)

b Plasma and leptomeningeal metastasis

Leptomeningeal meatstasis

		Yes	No	Total
**Plasma**	**Negative**	**4**	**15**	**19**
	**Positive**	**0**	**16**	**16**
**Total**		**4**	**31**	**35**

	Value	df	Asymptotic Significance (2-sided)
Pearson Chi-Square	3.803	1	0.051
Likelihood Ratio	5.320	1	0.021
N of Valid Cases	35		

p= 0.051 (no significance between positiv LB in plasma and leptomeningeal metastasís)

c CSF and Necrosis

Necrosis

		Yes	No	Total
**CSF**	**Negative**	**5**	**14**	**19**
	**Positive**	**5**	**11**	**16**
**Total**		**10**	**25**	**35**

	Value	df	Asymptotic Significance (2-sided)
Pearson Chi-Square	0.104	1	0.748
Likelihood Ratio	0.103	1	0.748
N of Valid Cases	35		

p= 0.748 (no significance between positiv LB in CSF and necrosis)

d Plasma and Necrosis

Necrosis

		Yes	No	Total
**Plasma**	**Negative**	**4**	**15**	**19**
	**Positive**	**6**	**10**	**16**
**Total**		**10**	**25**	**35**

	Value	df	Asymptotic Significance (2-sided)
Pearson Chi-Square	1.151	1	0.283
Likelihood Ratio	1.152	1	0.283
N of Valid Cases	35		

p= 0.283 (no significance between positiv LB in plasma and necrosis)

Supplementary Figure 8: Longitudinal monitoring of LB_MUV_8

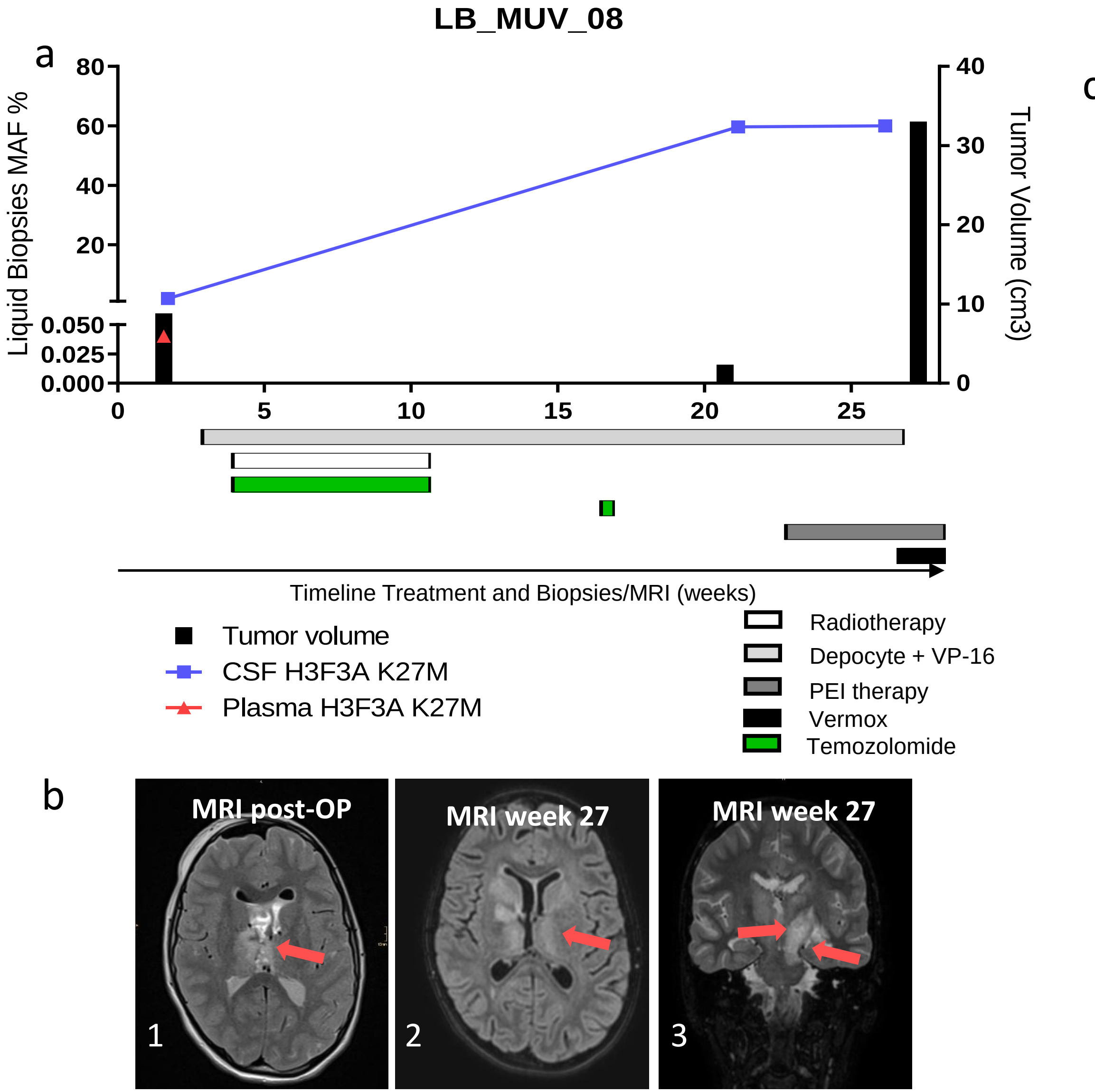

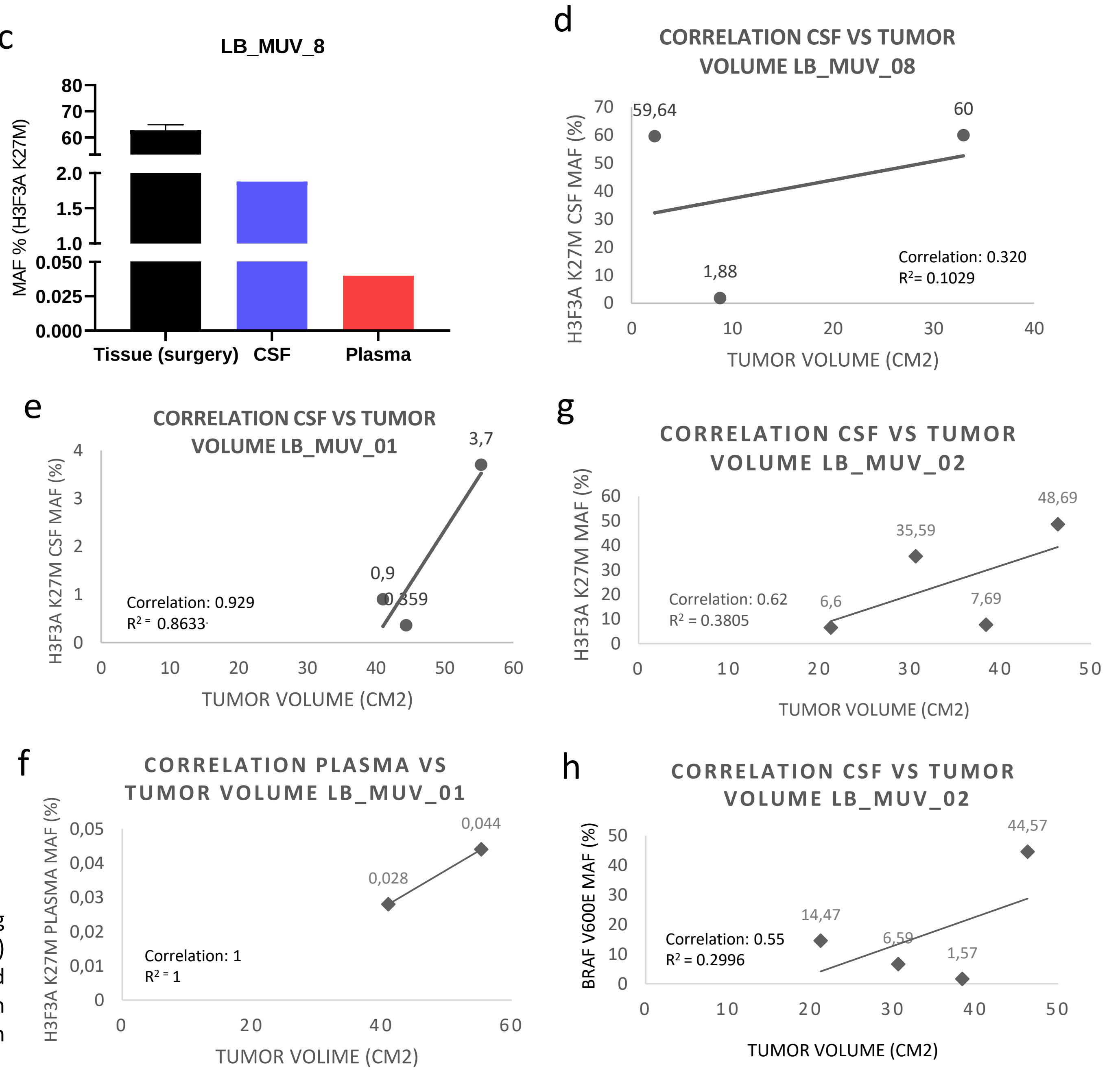

Supplementary Figure 8: Longitudinal monitoring of LB_MUV_08, treatment history including MAF of liquid biopsies and tumor volumes obtained from MRI (cm2) of a) LB_MUV_08 and b) matched MRI images (1+2 are axial FLAIR; image 3 is a coronal T2-weighted sequence) to liquid biopsy MAF detection. Red arrow marks the tumor spread. c) Initial MAF of H3F3A K27M in tissue vs initial liquid biopsy samples CSF pre-surgery and plasma pre-surgery. D-h) Correlation plots of LB_MUV_01, LB_MUV_02 and LB_MUV_08.

Supplementary Figure 9: Longitudinal monitoring in CSF and plasma vs MRI tumor volume

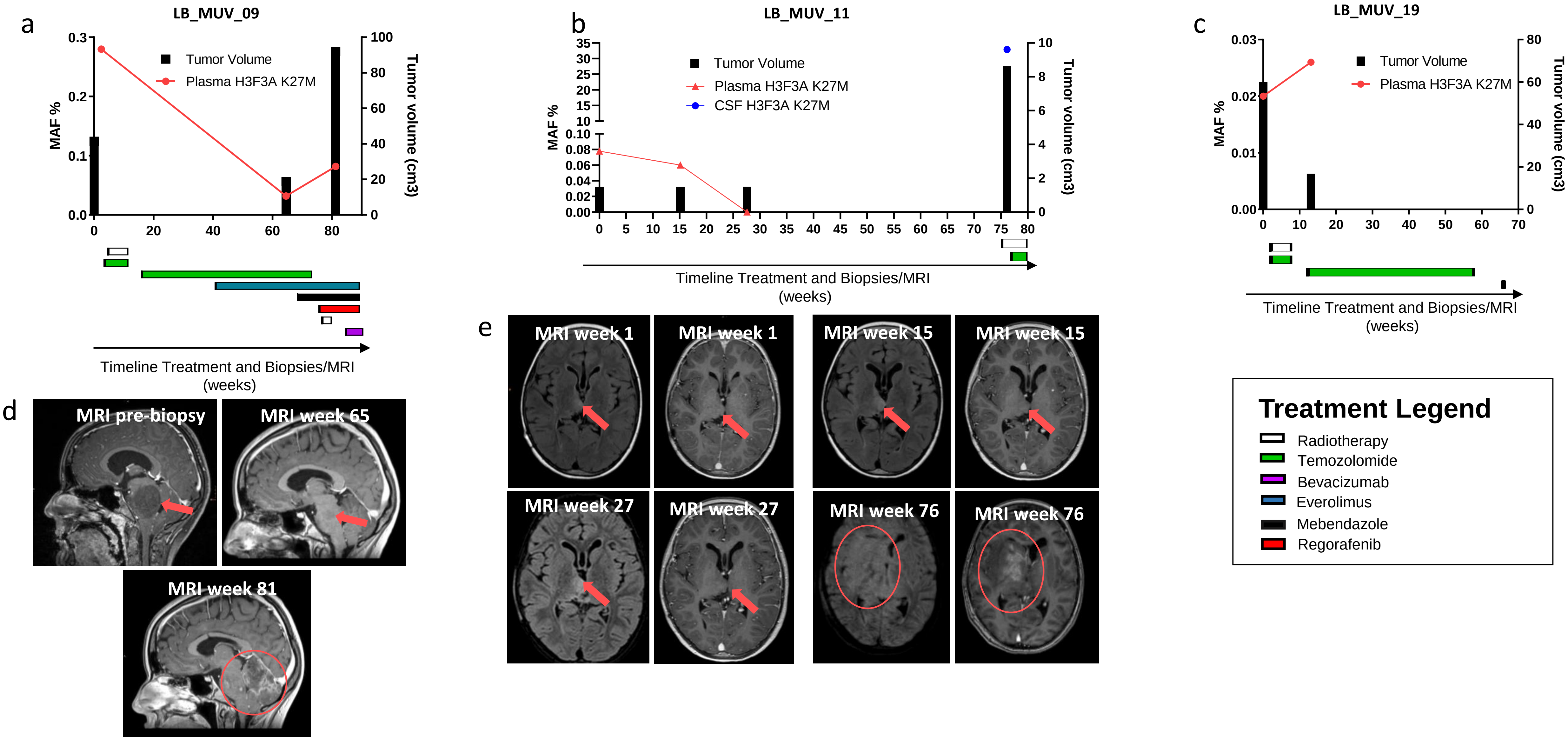

Supplementary Figure 9: Longitudinal monitoring of a) LB_MUV_09, b) LB_MUV_11, c) LB_MUV_19,. The graphs represent MRI tumor volumes (black bar) and the corresponding LB (red plasma, blue CSF). Below each graph, the timeline including treatment strategies is displayed. d) LB_MUV_09 matched MRI images (sagittal T1-weighted post-contrast sequence) e) LB_MUV_11 matched MRI images (axial FLAIR (left for each pair) and T1 weighted post-contrast sequence (right). Red arrow and circles marks the tumor spread.

Supplementary Figure 10: Longitudinal monitoring in CSF and plasma vs MRI tumor volume

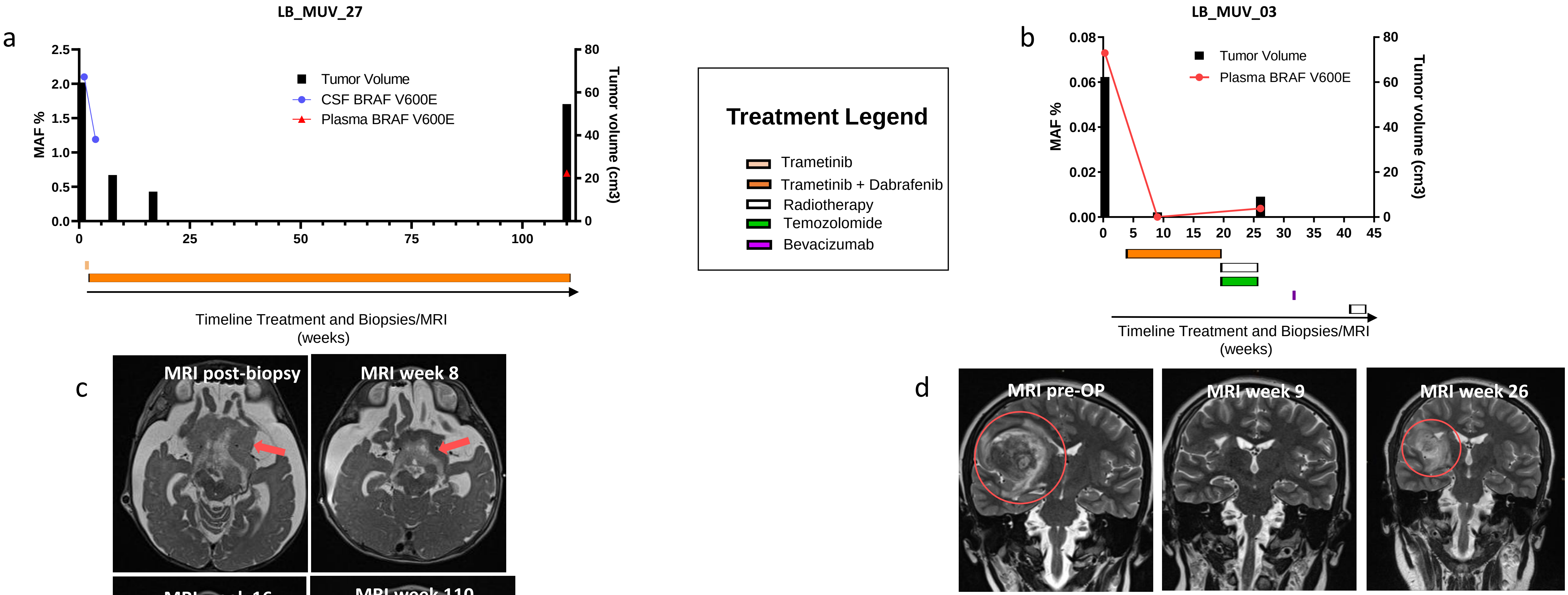

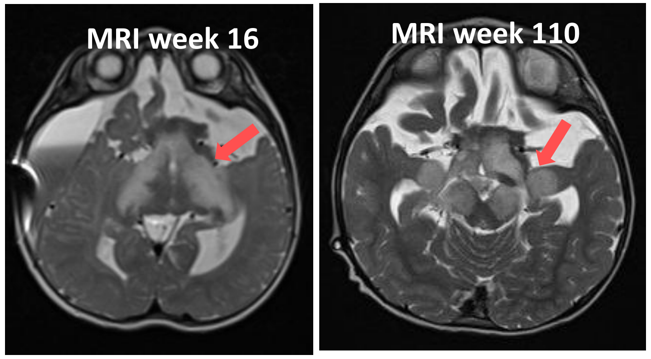

Supplementary Figure 10: Longitudinal monitoring of a) LB_MUV_27, b) LB_MUV_03. The graphs represent MRI tumor volumes (black bar) and the corresponding LB (red plasma, blue CSF). Below each graph, the timeline including treatment strategies is displayed. c) matched MRI images of LB_MUV_27, (axial T2 weighted sequence) and d) matched LB_MUV_03 (coronal T2 weighted sequence). Red arrow and circles marks the tumor spread
